# Pulmonary Mucormycosis in Chronic Lymphocytic Leukemia and Neutropenia

**DOI:** 10.1155/2018/2658083

**Published:** 2018-02-19

**Authors:** Izza Mir, Sijan Basnet, David Ellsworth, Elan Mohanty

**Affiliations:** Department of Medicine, Reading Health System, 420 S. Fifth Avenue, West Reading, PA 19611, USA

## Abstract

Pulmonary mucormycosis is a rare life-threatening fungal infection associated with high mortality. We present the case of a 61-year-old man with history of chronic lymphocytic leukemia who presented with fever and cough, eventually diagnosed with pulmonary mucormycosis after right lung video-assisted thoracoscopic surgery. The patient was successfully treated with amphotericin B and right lung pneumonectomy; however, he later died from left lung pneumonia.

## 1. Introduction

Pulmonary mucormycosis is a rare but life-threatening, rapidly progressive infection that occurs in immunocompromised patients with mortality as high as 76% that is 10–50 times less likely than aspergillus or candida infection [1–3]. Early clinical diagnosis can be delayed in the setting of concomitant infections such as pneumonia and bacteremia. However, prompt diagnosis is critical to initiate early aggressive management. We report the case of a patient with chronic lymphocytic leukemia (CLL) with resultant neutropenia undergoing chemotherapy diagnosed with pulmonary mucormycosis.

## 2. Case Description

A 61-year-old man with past medical history of sarcoidosis, deep vein thrombosis on rivaroxaban, and CLL treated with bendamustine and rituximab complicated by neutropenia presented to the outpatient office with (four) days of fevers and scant hemoptysis. On physical exam, patient had temperature of 38.4°C, blood pressure of 128/60 mm Hg, respirations of 18 per minute, heart rate of 92 beats per minute, and oxygen saturation of 96% in room air. Lung sounds were diminished bilaterally. His white blood cell count was 88,200/*µ*L with absolute neutrophil count 1900/UI (2.15%), hemoglobin level 7.4 g/dL (decreased from 8.8 g/dL three weeks prior), and platelet count 291,000/*µ*L. Chest radiograph revealed a large mass-like consolidation in the right upper lobe, which was new compared to computerized tomography (CT) imaging two months prior.

**Figure 1:**
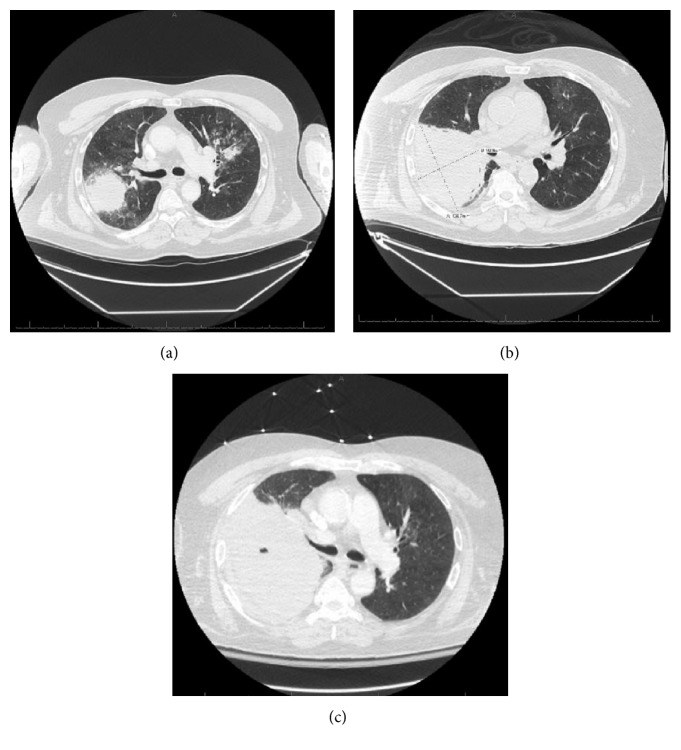
CT chest from day of admission (a), day of fine needle biopsy (b), and prior to VATS (c).

The patient was transported to the hospital, admitted for sepsis secondary to community-acquired pneumonia, and initiated on intravenous broad-spectrum antibiotics. CT chest was obtained due to concern for hemorrhage on ibrutinib. It revealed a mass-like consolidation in the right upper lobe along with diffuse nodules and multifocal airspace disease suspicious for invasive aspergillosis (Figure 1). Aspergillus antibody was obtained; however, empiric antifungal therapy was deferred while blood cultures grew *S. pneumoniae*. The patient failed to improve clinically prompting bronchoscopy evaluation, which identified purulent airway secretions. Bronchial aspirate cytology was unrevealing, and specimens were sent for further microbiology testing. The patient continued to decline. CT-guided needle biopsy of the consolidation established presence of scattered Gomori methenamine silver (GMS) and periodic acid-Schiff (PAS) positive structures suggestive of fungal etiology. So, voriconazole was started.

**Figure 2:**
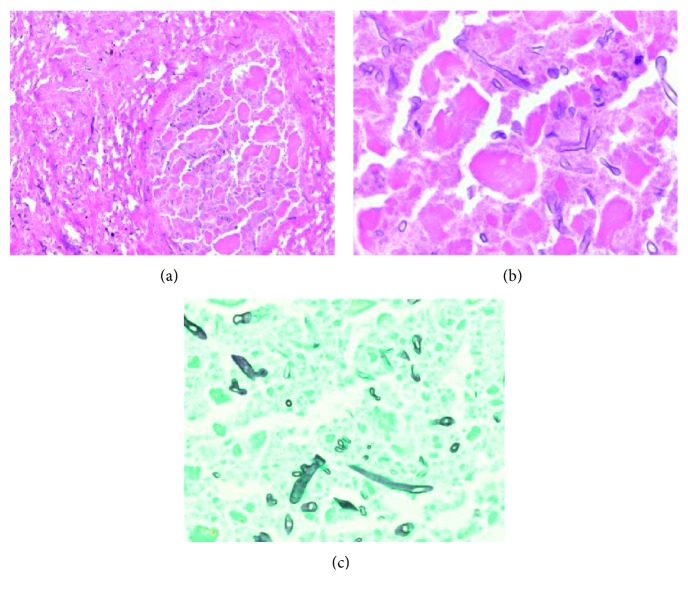
Histologic sections of pulmonary parenchyma showing prominent necrosis and angioinvasion by fungal species. Fungal hyphal forms noted in lumen and wall of pulmonary vessel (a). Intravascular broad and ribbon-like hyphal forms with right-angle branching and absence of septations, morphologically consistent with zygomycetic infection (hematoxylin/eosin stain (b); GMS staining (c)).

Repeat chest CT demonstrated worsening airspace disease, and the patient underwent video-assisted thoracoscopic surgery (VATS) with right upper and middle lobe wedge resection. VATS revealed severe dense adhesions, thickened and necrotic pleura, and the lung was found to be consolidated, hard, and hepatized with necrosis (Figure 2). Preliminary cultures were negative but eventually grew *Rhizopus* species. Since voriconazole is not effective against *Rhizopus*, it was discontinued and the patient was started on amphotericin B liposomal 5 mg/kg/24 hours. Six days after VATS, histopathologic analysis revealed presence of nonseptate fungal hyphae with right-angle branching suggestive of mucormycosis. Patient failed to improve after VATS; thus, patient underwent right pneumonectomy with subsequent clinical improvement. He was initially discharged to rehab eight days after pneumonectomy (43 days after admission) with plan for a 14-day course of amphotericin B after pneumonectomy followed by initiation of posaconazole as recommended per infectious disease specialist. Unfortunately, the patient returned to the hospital eleven days after discharge with complications of wound dehiscence and bronchocutaneous fistula formation that grew *Rhizopus*. Thus, patient's amphotericin B course was prolonged to a total of 46 days after pneumonectomy after which he was transitioned to posaconazole. This gentleman unfortunately suffered from further wound complications, VRE infection, and was unable to return home, and discharged to a nursing facility several months later. Patient returned to the hospital from nursing home with left lung pneumonia positive for rhinovirus-enterovirus and eventually passed away seven months later due to complications during that admission.

## 3. Discussion

Mucormycosis can take several forms including rhinocerebral, pulmonary, cutaneous, gastrointestinal, and disseminated disease [2]. Risk factors include diabetes mellitus, hematologic malignancy, receiving hematopoietic stem cell transplant, deferoxamine therapy, injection drug use, and trauma or burns [1–4]. Pulmonary infection is most commonly found in patients with hematologic malignancy, as was the case in our patient [1, 2, 5]. Additional risk factors for pulmonary infection include male gender, neutropenia, chronic steroid use, underlying rheumatologic disorders, and voriconazole prophylaxis in patients undergoing chemotherapy [2, 5–7]. Bendamustine and rituximab have been associated with impaired lymphocyte recovery in patients with CLL, and both agents have also been associated with an increased risk of serious opportunistic infections [8, 9]. Thus, in the case of our patient, these chemotherapy agents may also have contributed to the development of mucormycosis.

The symptoms are typically nonspecific even in later stages of infection, and co-infection can further complicate diagnosis. Infection most commonly presents as acute-onset high fever with nonproductive cough, dyspnea, or chest pain that is unresponsive to broad-spectrum antibiotics [2, 4, 7].

Radiological manifestations are mostly nonspecific, and disease tends to impact the upper lobes [4]. Findings on imaging that suggest pulmonary mucormycosis are greater than ten nodules, presence of pleural effusion, and a reverse halo sign on CT chest [2, 10, 11]. Bourcier et al. found that the presence of the reverse halo sign is more common in neutropenic patients with pulmonary mucormycosis, enough for them to suggest initiation of mucormycosis antifungal therapy based on the presence of this sign [10]. Sputum secretions and broncheoalveolar lavage cultures are notoriously insensitive for detection of mucormycosis, and even cultures from fine needle aspiration often fail to grow [4, 5]. The sensitivity can be improved by placing tissue sections directly on a culture plate without prior grinding or homogenizing the material, as it can disrupt the fungal structure [4, 5]. Colonies can take three to five days to grow. Diagnosis is achieved by demonstrating hyphae with variable width (from 6 to 25 *µ*m), zero or sparse septations, irregular ribbon-like appearance, and nonpigmented and wide-angle bifurcations including 90-degree angles. PAS and GMS stains must be used to properly visualize the hyphae [5]. Mucorales PCR has been shown to be useful for confirmation of the diagnosis of mucormycosis; however, it requires further investigation as a method of improving detection of mucormycosis in the clinical setting [12].

### 3.1. Management

Pulmonary mucormycosis has a poor prognosis. Patients who undergo combination of pharmacological and surgical management have been shown to do better compared to either treatment alone [4, 12, 13]. The drug of choice for initial therapy is amphotericin B while posaconazole or isavuconazole can be used for step-down therapy [14, 15]. Correction of underlying predisposing conditions, such as acidosis or neutropenia, and discontinuation of steroids or iron-chelating agents is required. Posaconazole has been reported as an effective salvage therapy in studies [14, 16]. Cornely et al. also showed that posaconazole improved survival and more effectively prevented invasive fungal infections than fluconazole or itraconazole in patients with hematologic malignancy undergoing chemotherapy [17]. Thus, to prevent severe mucor infections, posaconazole could potentially be considered for prophylaxis in patients with hematologic malignancy, especially those undergoing chemotherapy who are at risk for neutropenia.

## 4. Conclusion

Pulmonary mucormycosis is a rare but potentially fatal fungal infection that occurs in immunocompromised patients, most commonly in those with hematologic malignancy [1, 4]. Early recognition is crucial for prompt initiation of pharmacological and surgical comanagement to improve high rates of associated morbidity and mortality [2, 12]. Due to its nonspecific presentation, diagnosis is often difficult. However, suspicion and early initiation of therapy should be considered in immunosuppressed patients, especially neutropenic patients with hematologic malignancy, who present with fever and cough that is unresponsive to broad-spectrum antibiotics.
